# Supplementary material for: Effect of Staphylococcus aureus Contamination on the Microbial Diversity and Metabolites in Wholewheat Sourdough
Source: Foods. 2022 Jul 1;11(13):1960. doi: 10.3390/foods11131960 (PMC9265278; doi:10.3390/foods11131960)
Supplement: Supplementary file 1 [file foods-11-01960-s001.zip › foods-1760849-supplementary.pdf]

Table S1. The metabolites (g/100g) of fresh dough (Flo), sourdough fermented for 16h (Co16) and sourdough artificial contaminated with *S. aureus* (10^2 cfu/g) fermented for 16h (S16)

No.	Metabolites	S16	Co16	Flo
1	Pyruvic acid	0.002±0.003	0.005±0.001	n.d.
2	Lactic acid	0.023±0.005^{a}	0.019±0.002^{a}	0.003±0.001^{b}
3	Glycolic acid	4.870±1.370^{a}	3.989±0.313^{a}	0.008±0.001^{b}
4	2-Ketobutyric acid	0.003±0.004	0.002±0.004	0.005±0.001
5	Maleimide	0.004±0.002	0.004±0.001	0.002±0.001
6	2-Keto-isovaleric acid	0.003±0.001	0.003±0.001	0.003±0.001
7	Oxalic acid	0.010±0.001^{a}	0.010±0.002^{a}	0.004±0.001^{b}
8	3-Hydroxypropionic acid	0.004±0.001	0.003±0.001	0.003±0.001
9	Lactamide	n.d.	0.002±0.001	0.001±0.001
10	Sulfuric acid	0.007±0.001	0.007±0.001	0.005±0.001
11	N-Methyl-DL-alanine	n.d.	0.001±0.001	n.d.
12	Methyl Phosphate	0.002±0.001	0.002±0.001	0.002±0.001
13	Norleucine	0.001±0.001	0.001±0.001	0.001±0.001
14	Malonic acid	0.005±0.001	0.004±0.001	0.005±0.001
15	Naphthalene	0.005±0.001	0.005±0.001	0.007±0.001
16	Valine	0.041±0.003^{a}	0.040±0.001^{a}	0.013±0.001^{b}
17	4-Hydroxybutyrate	0.002±0.001	0.002±0.001	0.002±0.001
18	Citraconic acid	0.001±0.001	0.001±0.001	0.002±0.001

19	Benzoic acid	0.002±0.001	0.002±0.001	0.002±0.001
20	Ethanolamine	0.019±0.001[a]	0.019±0.001[a]	0.006±0.001[b]
21	Phosphate	1.173±0.148[a]	1.126±0.079[a]	0.122±0.006[b]
22	Glycerol	0.238±0.065	0.199±0.015	0.246±0.010
23	Cyclohexane-1,2-diol	n.d.	n.d.	0.003±0.001
24	N-cyclohexylformamide	0.018±0.003[b]	0.016±0.001[b]	0.022±0.001[a]
25	Nicotinic acid	n.d.	0.001±0.001	n.d.
26	Isoleucine	0.015±0.001[a]	0.015±0.001[a]	0.003±0.001[b]
27	Proline	0.041±0.004[a]	0.042±0.003[a]	0.002±0.001[b]
28	Maleic acid	0.001±0.001	0.001±0.001	n.d.
29	Glycine	0.052±0.007[a]	0.057±0.001[a]	0.011±0.001[b]
30	Succinic acid	0.186±0.020[a]	0.144±0.014[b]	0.028±0.001[c]
31	1,4-Cyclohexanedione	0.001±0.001	0.002±0.001	0.002±0.001
32	D-Glyceric acid	0.008±0.001	0.008±0.001	0.008±0.001
33	Uracil	0.006±0.001	0.006±0.001	n.d.
34	Fumaric acid	0.001±0.001[b]	0.002±0.001[b]	0.013±0.001[a]
35	Serine	0.002±0.001	0.003±0.001	0.001±0.001
36	3-Hydroxynorvaline	0.005±0.001	0.006±0.001	0.004±0.001
37	Pelargonic acid	0.002±0.001	0.001±0.001	0.001±0.001
38	L-Allothreonine	0.005±0.001	0.006±0.001	0.001±0.001
39	Resorcinol	0.001±0.001	0.001±0.001	0.001±0.001
40	Thymine	0.001±0.001	0.001±0.001	n.d.

41	β-Alanine	0.001±0.001	0.001±0.001	0.001±0.001
42	Erythrose	0.002±0.001	0.002±0.001	0.002±0.001
43	3-Aminoisobutyric acid	0.003±0.001	0.002±0.001	0.003±0.001
44	Aminomalonic acid	0.001±0.001	0.002±0.001	0.001±0.001
45	L-Malic acid	0.001±0.001[b]	0.003±0.001[b]	1.154±0.034[a]
46	Threitol	0.001±0.001	0.001±0.001	0.001±0.001
47	4-Acetamidobutyric acid	0.002±0.001	0.002±0.001	0.002±0.001
48	Aspartic acid	0.034±0.003[a]	0.037±0.002[a]	0.028±0.001[b]
49	Methionine	0.002±0.001	0.002±0.000	n.d.
50	Oxoproline	0.064±0.003[b]	0.065±0.002[b]	0.114±0.005[a]
51	4-Aminobutyric acid (GABA)	0.235±0.016[a]	0.241±0.017[a]	0.024±0.001[b]
52	L-glutamic acid	0.003±0.001	0.001±0.001	0.005±0.001
53	Maleamate	0.001±0.001	0.001±0.001	0.001±0.001
54	Threonic acid	0.001±0.001	0.001±0.001	n.d.
55	Phenylalanine	0.022±0.001[a]	0.021±0.001[a]	0.005±0.001[b]
56	N-Methyl-L-glutamic acid	0.001±0.001	0.001±0.001	0.002±0.001
57	2-hydroxy-3-isopropylbutanedioic acid	0.008±0.001	0.008±0.001	0.001±0.001
58	Digitoxose	0.003±0.001	0.003±0.001	n.d.
59	D-erythronolactone	0.003±0.001	0.004±0.001	0.004±0.001

60	Glutamic acid	0.002±0.001	0.002±0.001	0.002±0.001
61	Thymidine	0.001±0.000	0.001±0.001	n.d.
62	Tartaric acid	n.d.	0.001±0.001	0.001±0.001
63	Toluenesulfonic acid	0.002±0.001	0.002±0.001	0.006±0.001
64	Creatine	n.d.	0.001±0.001	0.001±0.001
65	Lyxose	0.034±0.001[a]	0.032±0.001[a]	0.001±0.001[b]
66	1,3-Diaminopropane	0.001±0.001	n.d.	n.d.
67	Xylose	0.751±0.128[a]	0.659±0.056[a]	0.014±0.001[b]
68	Asparagine	0.007±0.001	0.009±0.001	0.007±0.001
69	Ribose	0.099±0.003[b]	0.106±0.002[a]	0.002±0.001[c]
70	Guanidinosuccinic acid	n.d.	0.001±0.001	n.d.
71	3-Ureidopropionate	0.002±0.001	0.002±0.001	0.002±0.001
72	Xylitol	0.006±0.001	0.006±0.001	0.006±0.001
73	Biuret	0.007±0.001	0.007±0.001	0.007±0.001
74	D-Arabitol	0.019±0.001[b]	0.020±0.001[b]	0.023±0.001[a]
75	Ribitol	0.002±0.002	0.002±0.001	0.002±0.001
76	Putrescine	0.013±0.001[a]	0.012±0.001[a]	0.002±0.001[b]
77	Aconitic Acid	0.010±0.001[a]	0.011±0.001[a]	0.006±0.001[b]
78	Glucose-1-phosphate	0.020±0.002[b]	0.022±0.002[b]	0.054±0.023[a]
79	D-glycerol 1-phosphate	0.014±0.001[a]	0.015±0.001[a]	0.008±0.001[b]
80	2-deoxy-D-glucose	0.001±0.001	0.001±0.001	0.001±0.001
81	2-Deoxy-D-galactose	0.001±0.000	0.002±0.001	n.d.

82	Azelaic acid	0.001±0.001	0.001±0.001	0.001±0.001
83	Shikimic acid	0.001±0.001	0.001±0.001	0.001±0.001
84	Citric acid	0.001±0.000[c]	0.005±0.001[b]	0.030±0.006[a]
85	Ornithine	0.002±0.001	0.002±0.001	0.001±0.001
86	Citrulline	0.002±0.001	0.002±0.001	0.001±0.001
87	N(α),N(α)-dimethyl-L-histidine	0.001±0.001	0.001±0.001	n.d.
88	1,5-Anhydroglucitol	0.002±0.001	0.002±0.001	n.d.
89	Tagatose	0.001±0.001	0.001±0.001	0.001±0.001
90	Glycocyamine	0.001±0.000	0.001±0.001	0.001±0.001
91	Dehydroascorbic Acid	0.003±0.001	0.003±0.001	n.d.
92	Quinic acid	0.002±0.001	0.002±0.001	0.002±0.001
93	Myristic Acid	0.001±0.001	0.002±0.001	0.002±0.001
94	Fructose	5.488±1.339[a]	5.225±0.432[a]	1.041±0.022[b]
95	Glucose	0.757±0.182[a]	0.649±0.042[a]	0.033±0.001[b]
96	Gluconic lactone	n.d.	n.d.	0.003±0.001
97	Mannose	0.057±0.014[c]	0.278±0.086[a]	0.134±0.003[b]
98	Galactose	0.014±0.014	0.006±0.005	n.d.
99	Tyramine	0.012±0.002	0.011±0.000	n.d.
100	Atrazine-2-hydroxy	0.005±0.003[b]	0.005±0.001[b]	0.018±0.001[a]
101	Lysine	0.025±0.002[a]	0.028±0.001[a]	0.002±0.001[b]
102	Sorbitol	1.010±0.231[a]	0.463±0.038[b]	0.027±0.002[c]

103	D-galacturonic acid	0.005±0.001	0.005±0.001	0.001±0.001
104	Tyrosine	0.041±0.004[a]	0.039±0.002[a]	0.007±0.001[b]
105	Pentadecanoic acid	n.d.	n.d.	0.001±0.001
106	Conduritol-β-epoxide	0.005±0.001	0.006±0.001	n.d.
107	Lipoic acid	0.001±0.002	0.001±0.001	0.001±0.001
108	Galactonic acid	0.001±0.001	0.001±0.001	0.001±0.001
109	Gluconic acid	0.001±0.001	0.001±0.001	0.003±0.001
110	Xanthine	0.002±0.002	0.002±0.002	n.d.
111	Glucosaminic acid	n.d.	n.d.	n.d.
112	Palmitic acid	0.221±0.043	0.261±0.061	0.218±0.012
113	N-Acetyl-β-D-mannosamine	n.d.	n.d.	0.004±0.001
114	N,N-dimethylarginine	0.001±0.000	0.001±0.000	0.001±0.001
115	Myo-inositol	2.044±0.356[a]	1.741±0.151[a]	0.166±0.011[b]
116	Uric acid	0.002±0.001	n.d.	0.002±0.001
117	Ferulic acid	0.001±0.001	n.d.	n.d.
118	trans-3,5-Dimethoxy-4-hydroxycinnamaldehyde	0.001±0.001	0.001±0.001	0.001±0.001
119	Glucoheptonic acid	0.017±0.001[a]	0.014±0.001[b]	n.d.
120	d-Glucoheptose	0.029±0.002[a]	0.020±0.001[b]	n.d.
121	Heptadecanoic acid	0.002±0.003	n.d.	n.d.
122	Isoxanthopterin	0.001±0.001	0.001±0.001	0.001±0.001

123	Octadecanol	0.003±0.001	0.004±0.001	0.003±0.001
124	1,4-Dihydroxy-2-naphthoic acid	0.001±0.001	0.001±0.001	0.001
125	β-Mannosylglycerate	n.d.	0.001±0.001	n.d.
126	Fructose-2,6-biphosphate	0.001±0.001	0.001±0.001	0.001±0.001
127	3-Indolepyruvic acid	0.008±0.007	0.007±0.006	0.013±0.003
128	Tryptophan	n.d.	0.036±0.063	0.076±0.015
129	Linoleic acid	0.131±0.006^{b}	0.112±0.011^{b}	0.234±0.006^{a}
130	Oleic acid	0.003±0.001	0.003±0.001	0.007±0.001
131	Linolenic acid	0.001±0.001	0.002±0.001	0.007±0.001
132	Elaidic acid	0.002±0.001	0.002±0.001	0.003±0.001
133	Stearic acid	0.098±0.008	0.113±0.021	0.102±0.002
134	Purine riboside	0.011±0.001	0.010±0.001	0.011±0.001
135	6-Phosphogluconic acid	0.011±0.001^{a}	0.011±0.001^{a}	0.001±0.001^{b}
136	cis-Gondoic acid	n.d.	n.d.	0.001±0.001
137	Arachidic acid	0.001±0.000	0.001±0.000	0.001±0.001
138	Cytidine-monophosphate	0.002±0.001	0.001±0.001	n.d.
139	Abietic Acid	0.001±0.001	0.001±0.001	n.d.
140	Uridine	0.002±0.001	0.002±0.001	0.002±0.001
141	DL-dihydrosphingosine	0.004±0.001	0.002±0.002	0.006±0.001
142	Salicin	0.014±0.001^{a}	0.011±0.002^{b}	0.010±0.001^{b}
143	Arbutin	n.d.	n.d.	0.001±0.001

144	1-Monopalmitin	n.d.	n.d.	0.001±0.001
145	Glutathione - H_2O	0.002±0.001	0.002±0.001	n.d.
146	Sucrose	0.037±0.005[c]	0.054±0.004[b]	0.218±0.015[a]
147	Lactose	0.345±0.124[a]	0.326±0.065[a]	0.009±0.001[b]
148	2-Monoolein	0.020±0.035	n.d.	0.001±0.001
149	Cellobiose	n.d.	0.002±0.002	n.d.
150	Trehalose	n.d.	0.005±0.009	0.003±0.001
151	7-Hydroxy-4-androstene-3,17-dione	0.001±0.000	0.001±0.001	n.d.
152	Cortexolone	0.004±0.004	0.004±0.001	n.d.
153	Guanosine	n.d.	n.d.	0.001±0.001
154	Maltose	0.078±0.023[a]	0.067±0.013[a]	0.013±0.002[b]
155	4-Androstene-3,17-dione	0.001±0.001	n.d.	n.d.
156	Sophorose	0.010±0.001[a]	0.007±0.001[b]	n.d.
157	Gentiobiose	0.006±0.002	0.004±0.001	n.d.
158	Melibiose	0.042±0.011[a]	0.028±0.003[b]	0.011±0.001[c]
159	Isomaltose	0.058±0.007[a]	0.041±0.003[b]	0.001±0.001[c]
160	Galactinol	0.034±0.005[b]	0.032±0.002[b]	0.110±0.008[a]
161	7-α-Hydroxycholesterol	0.001±0.000	0.001±0.001	n.d.
162	Trehalose-6-phosphate	0.013±0.022	0.015±0.019	n.d.
163	5-Dihydrocortisone	n.d.	0.001±0.001	n.d.
164	Raffinose	0.002±0.002	0.003±0.001	n.d.

165	Kestose	0.353±0.017[c]	0.422±0.034[b]	0.933±0.166[a]
166	Cholestane-3,5,6-triol, (3β ,5α ,6β)-	0.026±0.005	0.034±0.008	0.030±0.002
167	Melezitose	0.005±0.001	0.005±0.001	n.d.
168	Maltotriose	0.026±0.001	0.024±0.001	n.d.
169	Prunin	0.026±0.006	0.017±0.007	n.d.

[a-c] Means in each row having different letters are significantly different ($p< 0.05$, n=3).

n.d., not detectable.
